# Dopaminergic Lesions of the Dorsolateral Striatum in Rats Increase Delay Discounting in an Impulsive Choice Task

**DOI:** 10.1371/journal.pone.0122063

**Published:** 2015-04-30

**Authors:** Stephanie E. Tedford, Amanda L. Persons, T. Celeste Napier

**Affiliations:** 1 Department of Pharmacology, Rush University, Chicago, Illinois, United States of America; 2 Department of Psychiatry, Rush University, Chicago, Illinois, United States of America; 3 Center for Compulsive Behavior and Addiction, Rush University, Chicago, Illinois, United States of America; Florey Institute of Neuroscience and Mental Health, The University of Melbourne, AUSTRALIA

## Abstract

Dysregulated dopamine transmission in striatal circuitry is associated with impulsivity. The current study evaluated the influence of dopaminergic inputs to the dorsolateral striatum on impulsive choice, one aspect of impulsive behavior. We implemented an operant task that measures impulsive choice in rats via delay discounting wherein intracranial self-stimulation (ICSS) was used as the positive reinforcer. To do so, rats were anesthetized to allow implanting of a stimulating electrode within the lateral hypothalamus of one hemisphere and bilateral dorsal striatal injections of the dopaminergic toxin, 6-OHDA (lesioned) or its vehicle (sham). Following recovery, rats were trained in a delay discounting task wherein they selected between a small ICSS current presented immediately after lever pressing, and a large ICSS current presented following a 0 to 15s delay upon pressing the alternate lever. Task acquisition and reinforcer discrimination were similar for lesioned and sham rats. All rats exhibited an initial preference for the large reinforcer, and as the delay was increased, preference for the large reinforcer was decreased indicating that the subjective value of the large reinforcer was discounted as a function of delay time. However, this discounting effect was significantly enhanced in lesioned rats for the longer delays. These data reveal a contribution of dopaminergic inputs to the dorsolateral striatum on impulsive choice behavior, and provide new insights into neural substrates underlying discounting behaviors.

## Introduction

Impulsivity is a general term that umbrellas many aspects of impulsive behavior, including suboptimal risk/reward, effort-based, and delay-based decision-making. Decision-making involves complex, reward-mediated processes that are sensitive to several weighted factors, including the probability, workload, and delay of a potential reward. Impulsive choice, a facet of delay-based decision-making, can be studied using delay discounting paradigms wherein subjects select between an immediate, often smaller reward, and a delayed, often larger reward. As the delay towards delivery of the large reward is increased, the subjective value of the reward often is decreased (i.e., discounted), and preference is shifted to the immediate, smaller reward. Impulsive choice is considered to reflect preference for the immediately available small reward without appropriate consideration for the larger, albeit delayed, reward. Impulsive choice is enhanced in numerous neuropathologies, including problem gambling and substance use disorders [1–7]. Thus, determining the neural anatomical substrates of impulsive choice is a critical aspect of understanding and subsequently treating these brain diseases.

The cortico-striatal pathway acts as an interface for reward-related circuitry in the striatum and executive functioning in the cortex. Both structures are necessary for the execution of appropriate decision-making; however, the particular contribution of each neural substrate is not well understood. The dorsolateral striatum (DLS) in rats, or the human homolog, the putamen, has historically been recognized for its involvement in motor function. Recent evidence also points to a role in decision-making, impulsivity and habitual/compulsive behaviors [8–13]. Several human studies using functional magnetic resonance imaging (fMRI) report dysregulated connectivity between the putamen and cortical brain structures in individuals who display impulsivity. Adolescents with internet addiction demonstrate reduced connectivity of the putamen with frontal, temporal and parietal cortices [14,15]. Cocaine-addicted subjects who exhibit impulsivity have reduced connectivity between the putamen and posterior insula and postcentral gyrus [14]. In addition, humans engaged in delay discounting tasks show activity in the putamen during delayed choice selections [9]. Altogether, these reports demonstrate a potential function for the DLS/putamen in decision-making and impulsive behavior; however, the precise role still remains to be determined.

The DLS/putamen receives significant dopaminergic projections from the substantia nigra pars compacta (SNpc). Accumulating evidence indicates that dysfunction in dopaminergic transmission contributes to impulsive behavior. Endogenous dopamine levels can be manipulated through various mechanisms including the metabolism and transport of dopamine in and out of the synaptic cleft. Polymorphisms in the DA transporter (DAT) gene 1, which regulates DA signaling by removing dopamine from the synapse, are correlated with impulsivity [16]. Reduced midbrain dopamine autoreceptor availability, which would promote dopaminergic neuronal firing, is also associated with the expression of impulsivity [17]. Humans treated with tolcapone, an inhibitor of catechol-O-methyl transferase which reduces dopamine metabolism, demonstrate reduced connectivity in the putamen during delay discounting tasks (as assessed using fMRI) [18]. Such observations have fueled a growing interest in dopaminergic circuits that may influence trait impulsivity and how the DLS/putamen may contribute to these behaviors. To our knowledge, no studies have directly assessed the role of dopaminergic inputs to the DLS/putamen on impulsive choice behavior. To investigate this issue, we implemented a novel delay discounting task in rats with dopaminergic lesions of the DLS.

Procedures for assessing delay discounting in rats commonly use food as the positive reinforcer. Satiety state can be a concern for appropriate interpretation of study outcomes with such protocols [19,20]; therefore, we utilized electrical stimulation of a brain ‘reward’ pathway (i.e., the medial forebrain bundle) as the positive reinforcer for the operant task. Several other features of ICSS made it attractive for our study. For example, rats prefer ICSS to food reinforcement [21,22]. ICSS results in faster acquisition of operant responding [23] and as satiety is not an issue, the rats can be tested several times in a day which reduces the number of days needed to generate stable responding, and the response stability is maintained for several weeks [24–26]. Previously, we reported on the use of ICSS for an operant task measuring probability discounting [24–26]; here we illustrate its utility in a delay discounting task. We hypothesize that decreased dopaminergic function in the DLS produced by intrastriatal injections of the neurotoxin 6-OHDA will influence impulsive choice behavior as measured in our delay discounting task.

## Materials and Methods

### Animals

Thirty male Sprague-Dawley rats, weighing 250–300g upon arrival from Harlan Laboratories (Indianapolis, IN), were housed in pairs under environmentally controlled conditions (temperature 23–25°C) in a 12h light-dark cycle (lights on at 7 A.M.). Food and water were available *ad libitum*. Experimental procedures were approved by the Rush University Medical Center IACUC, and rats were handled in accordance with established procedures in the *Guide for the Care and Use of Laboratory Animals* (National Research Council, Washington, DC).

### Procedures for intracerebral injections of the dopaminotoxin and implanting stimulation electrodes

Procedures followed our previously published protocols [26] and are further detailed in the Supporting Information. In brief, rats were pretreated with desipramine-HCl (25mg/kg as the salt, Sigma-Aldrich, St Louis, MO) 30min prior to isoflurane-induced anesthesia. A two 33-gauge cannulae assembly was lowered into the DLS of both hemispheres (1.0mm anterior to Bregma; ±3.4mm lateral to midline and 4.7mm below the skull surface [27]), and 6-OHDA (7.5μg as the salt, Sigma-Aldrich) (referred to as lesioned) or its 0.2% ascorbic acid vehicle (sham) was infused as 0.2μl/min for a total volume of 2μl per side. A single bipolar stimulating electrode (MS303/3-B/SPC; Plastics One, Roanoke, VA) was stereotaxically lowered to the lateral hypothalamus of the right hemisphere (-2.6mm posterior to Bregma; +1.8mm lateral to midline and -8.4mm from skull surface [27]). The electrode was secured to the skull with stainless steel screws and dental acrylic (referred to as the head-stage). The incision was sutured and rats were allowed to recover for a minimum of five days before behavioral testing began.

### Behavioral tasks

We used the forelimb adjustment step test to provide a behavioral readout of the 6-OHDA-induced lesion, per our previously published protocol [26]. In brief, the rats’ rear legs and one forepaw were held to the body so that the remaining forepaw was free to adjust their weight when moved across a bench top for 0.9m over 5s. Three trials were taken per session. Right and left forepaw stepping in the adduction and abduction direction show similar effects [26], and we report here the average score determined for the right forepaw in the abduction direction.

ICSS followed our published protocols [24–26]. Operant procedures used standard chambers for rats purchased from Med-Associates, Inc. (St. Albans, VT) and were equipped with two retractable levers, a stimulus light located above each lever and a central house light. The opportunity to receive brain stimulation (BrS) was indicated by illuminating the house light and extending lever(s) for the rats to press. Following each lever press, a stimulus light above the selected lever was illuminated for 500ms and electrical current was delivered to the intracranial electrode by constant current stimulators (PHM-152/2 Dual programmable ICSS stimulators, Med-Associates).

A delay discounting paradigm using ICSS as the positive reinforcer was implemented to measure impulsive choice. To our knowledge, this is the first time that this approach has been used; therefore, details of its application and of each of the identified benchmarks (i.e., Phases) are included in the Supporting Information. A brief overview is provided here:

#### Phase 1, shaping

A single lever was extended inside the operant chamber. BrS was delivered upon forward movement toward the direction of the extended lever. BrS was delivered in 200μs biphasic square wave pulses delivered at 100Hz for 500ms, starting at 100μA and adjusted in 20μA increments based upon each rat’s behavior.

#### Phase 2, fixed ratio-1 (FR-1) reinforcement

Using the current intensity that produced high rates of lever pressing in Phase 1, rats were trained in a FR-1 schedule (20min) of BrS reinforcement with continuous lever extension to establish stable (>8 lever presses/min) lever pressing responses.

#### Phase 3, delay discounting task

Upon lever pressing, one lever delivered the small, immediate reinforcer (SR; 50Hz) and the other delivered a large reinforcer (LR; 160Hz) that was given after a delay ranging between 0–15s. Both levers were retracted immediately following selection. The current frequencies used for the SR and LR were selected based upon a BrS current frequency *vs*. lever pressing rate function. A subset of rats were tested wherein it was validated that there was a positive relationship between the magnitude of the stimulation and the number of lever presses (S1 Fig). According to these frequency curves, 50Hz was located in the lower third of the curve that elicited stable, although moderate, lever pressing and 160Hz was consistently close to frequencies that elicited maximal lever pressing (detailed in the Supporting Information). We have previously determined using an ICSS-mediated probability discounting paradigm, that stimulation frequencies near 50Hz are clearly distinguishable from ‘no reward’ in a discrimination test [25,26].

**Fig 1:**
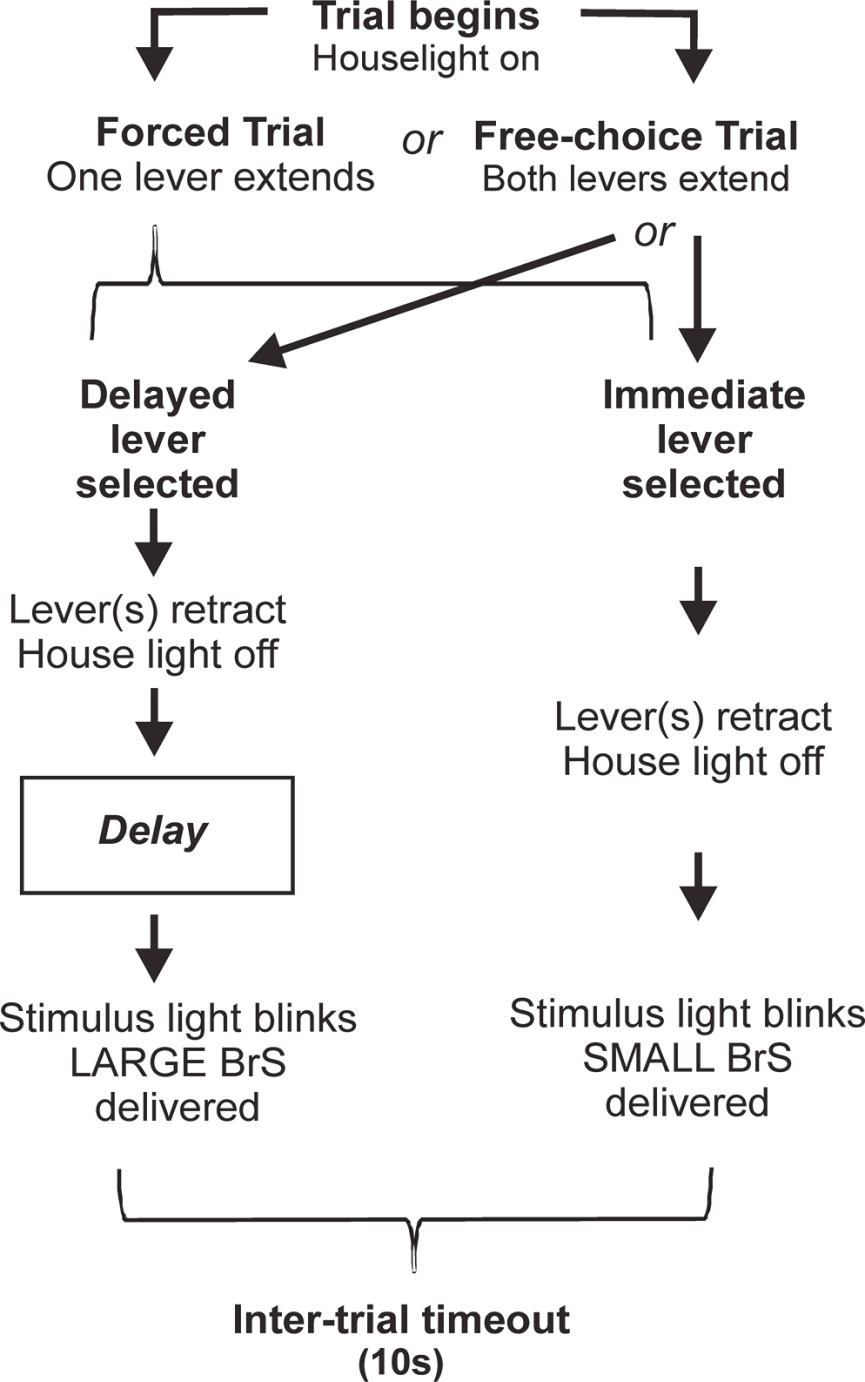
Flow chart showing contingencies for the delay discounting task. Definitions: LR, large reinforcer; SR, small reinforcer; BrS; brain stimulation. Rats are trained in forced trials (only one lever extended) to learn the contingencies of each lever, i.e., which lever delivers the small reinforcer (SR; 50Hz) presented immediately after a lever press, and which lever delivers the large reinforcer (LR; 160Hz) after various time intervals following a lever press. Free-choice trials allow the rat to make a selection between levers based on their individual preference. If no selection is made within 10s, the lever(s) are withdrawn and a 10s inter-trial timeout begins.

Each discounting session consisted of 3 blocks of 10 forced and 10 free-choice trials. During forced trials, only one lever was extended allowing rats to learn the contingencies of that lever (i.e., whether it was associated with the SR or the LR). During free-choice trials, both levers were extended and rats were allowed to select between the two options. Failure to select a lever within 10s was scored as an omission, and this resulted in retraction of the lever(s). The contingencies associated with each lever were varied between sessions to assure a lack of bias for one lever. The delays preceding delivery of the LR were 0, 3, 5, 8, 12 and 15s (Fig 1). Test sessions were conducted with only one delay at a time. Following stable behavior (<20% variability over three consecutive sessions), rats were advanced to the next delay in ascending order (rationale provided in Supporting Information). Data from free-choice trials of each block were analyzed to determine a baseline free-choice ratio (i.e., the number of selections made for the LR divided by total selections x 100) *vs*. delay. If in a block, there were >50% omissions from the free-choice trials (i.e., >5 of 10 trials tested), data from that block were excluded from subsequent analysis.

### Verification of electrode placement and lesion profile

Upon completion of the behavioral assessments, the rats were deeply anesthetized. The brains were removed, and sliced into 40μm coronal sections. The location of the stimulation electrode tip was verified by at least two observers in cresyl violet stained sections. The lesion produced by intra-striatal injections of 6-OHDA was determined using immunohistochemical (IHC) staining for tyrosine hydroxylase (TH) [26] with a primary antibody (1:10,000; ImmunoStar, Hudson, WI) and a biotinylated secondary antibody (horse anti-mouse, rat adsorbed, 1:200; Vector Laboratories, Burlingame, CA). Immunostaining was visualized using 0.5% 3,3-diaminobenzidinetetrachloridedihydrate (Sigma-Aldrich) with 1% H_2_O_2_. Lesion extent within the striatum was characterized by a near absence of TH-like staining as agreed upon by at least two observers. The number of TH^+^ soma within the SNpc was quantified using stereological approaches (see Supporting Information). The total number of TH^+^ cells and the volume of tissue were divided to obtain a density measurement expressed as total number/mm^3^ for each section and then averaged for five sections per rat.

### Statistical analyses

Statistical analysis conducted on data collected during the discounting paradigm was assessed in rats which performed throughout the entire paradigm with the same delay presentation history (i.e., lesioned, n = 9; sham, n = 6). To determine if task acquisition differed between the two treatment groups (i.e., lesioned and shams), data collected from phase 1 (shaping) and phase 2 (FR-1) of the discounting paradigm were analyzed using a Student’s *t*-test. The free-choice ratio collected during phase 3 (delay discounting) was analyzed using a two-way repeated measures analysis of variance (rmANOVA) with lesion status and delay as factors. A *post hoc* Newman-Keuls provided individual comparisons. Forelimb stepping deficits were analyzed using a two-way rmANOVA with lesion status and time as factors. Stereological assessments were analyzed using a Student’s *t*-test. Significance was accepted at *p*<0.05 and data are presented as the mean+SEM.

## Results

### Consequences of intra-DLS injections of 6-OHDA

**Fig 2:**
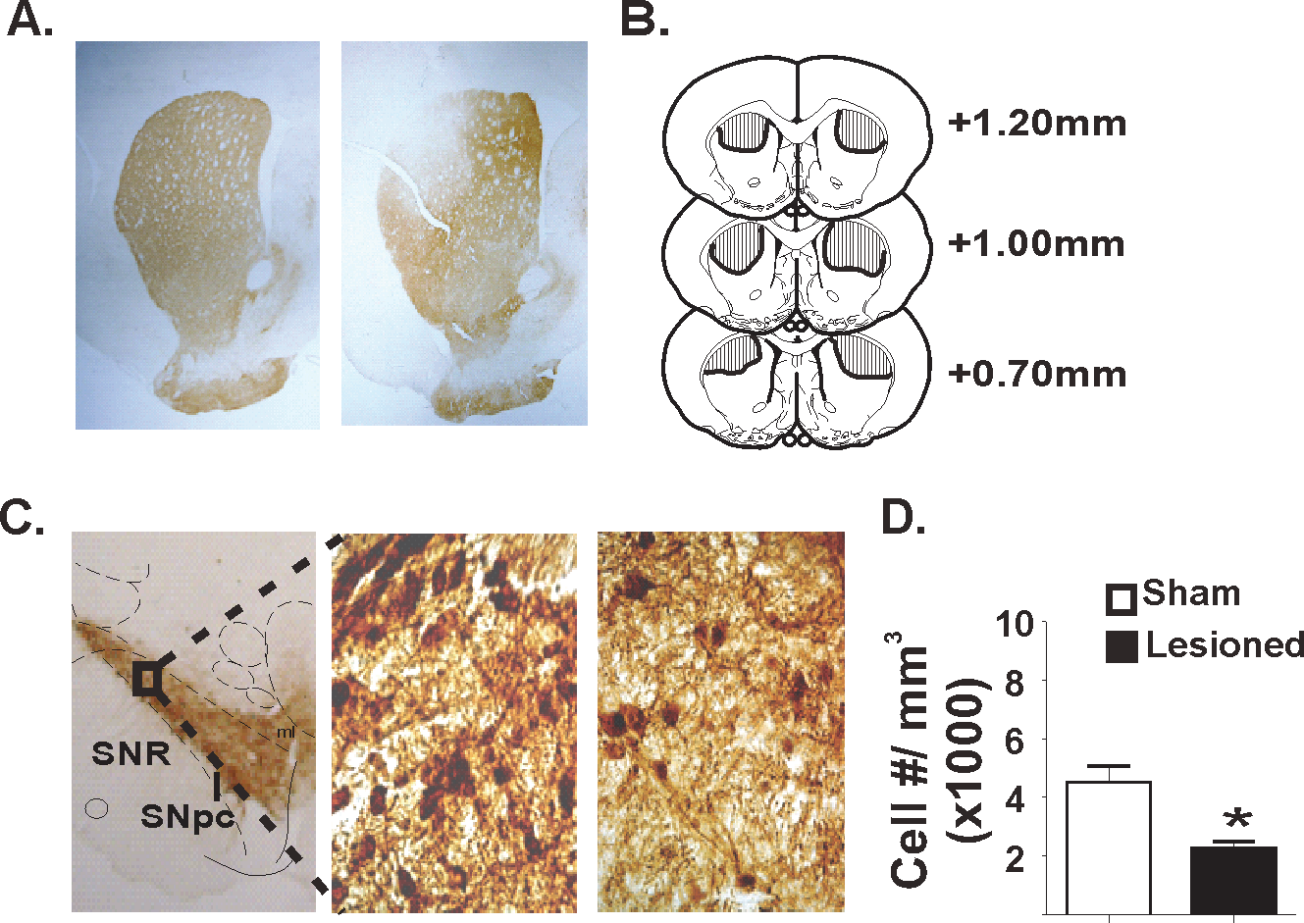
Lesion extent following intra-striatal injections of 6-OHDA. **(A)** Representative photomicrographs of tyrosine hydroxylase immunoreactivity (TH-ir) at the level of the DLS (~1.0mm AP from bregma) in one hemisphere. Compared with sham (vehicle-injected; left), 6-OHDA reduced staining in the DLS at 90 days (right) after treatment. **(B)** DLS lesions were targeted at +1.0mm from bregma. Tissue sections were outlined by two observers and the lesion extent was mapped to standard neuroanatomical plates. Lesion extent remained within 0.3mm of the target location. **(C)** Quantification of TH-ir in the SNpc. The left panels show low magnification photomicrographs of a SNpc coronal section in a sham (left) rat. This unilateral representation of the lesion was collapsed onto a neuroanatamoical plates (~5.80mm from bregma). The outlined box represents the location of the photomicrographs for the adjacent high magnification photomicrographs taken from a sham (middle) and lesioned (right) rat. **(D)** DLS lesions decreased cell counts in the ventrolateral region of the SNpc. Group means + SEM of stereological cell counts of TH-ir+ cells in the SNpc, (Student’s *t*-test, * p < 0.05) compared with sham controls. Neuroanatomical plates were provided by Paxinos and Watson (1998). SNR, Substantia nigra pars reticulata; SNpc, Substantia nigra pars compacta.

**Fig 3:**
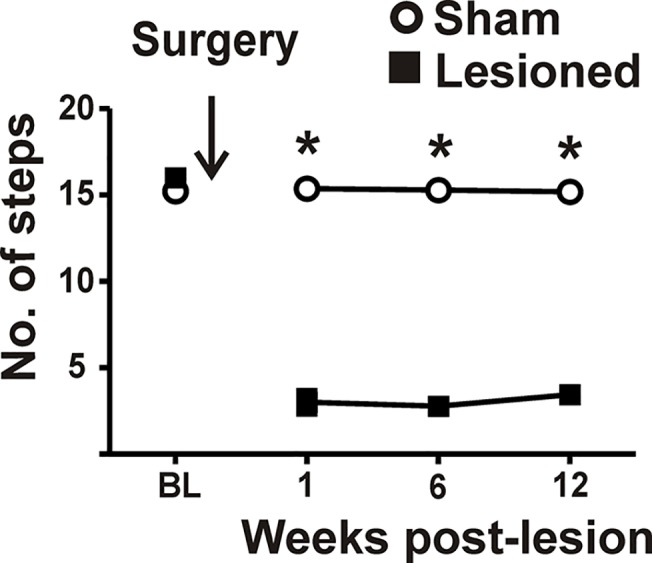
Stepping deficits subsequent to injecting 6-OHDA into the dorsolateral striatum. One day prior to surgery, wherein 6-OHDA was infused into the DLS, (Baseline; BL), and 1, 6, and 12 weeks following striatal 6-OHDA (n = 6) (or vehicle; i.e., shams n = 9) injections, rats were given a forelimb adjustment step test. Sham rats maintained normal stepping; however, 6-OHDA treated rats showed motor stepping deficits throughout the timeframe of the study. Shown are data collected from the right forepaw in the abduction direction, as mean±SEM steps/36”/5s (two way rmANOVA; *post hoc* Newman-Keuls, * p<0.01). Error bars do not extend beyond the size of the symbols.

Fig 2 illustrates the extent of TH immunoreactivity (TH-ir) 3 months following a vehicle (sham) or 6-OHDA injection into the DLS. There was a marked reduction in TH-ir in the DLS after 6-OHDA. In contrast, TH-ir in the DLS of shams was similar to surrounding tissue. The ventro-lateral region of the SNpc is the primary site of origin for dopaminergic neurons that project to the DLS. A subset of rats (Lesioned, 6; Sham, 6) were used to determine the effects of 6-OHDA-induced lesions of the DLS on TH-ir^+^ cell counts in the SNpc. Rats with intra-DLS injections of 6-OHDA displayed a significant decrease (~51%) in TH-ir^+^ cells within this nigral subregion compared to shams (*t*_(10)_ = 3.8, *p* = 0.003), indicating that lesions of the DLS caused retrograde loss of dopaminergic cell bodies located in the SNpc. To demonstrate a functional consequence of the 6-OHDA-induced lesions, rats were tested in the postural adjustment task (Fig 3). Baseline (pre-surgery) stepping was similar for all rats. 6-OHDA-treated rats showed a significant reduction in stepping, as assessed throughout the twelve weeks of behavioral testing (treatment [*F*_1,13_ = 391.99, *p*<0.001], time [*F*_3,39_ = 53.72, *p*<0.001] and interaction [*F*_3,39_ = 36.32, *p*<0.001]). There was no change in stepping for sham controls.

### ICSS and delay discounting protocols

#### ICSS

**Fig 4:**
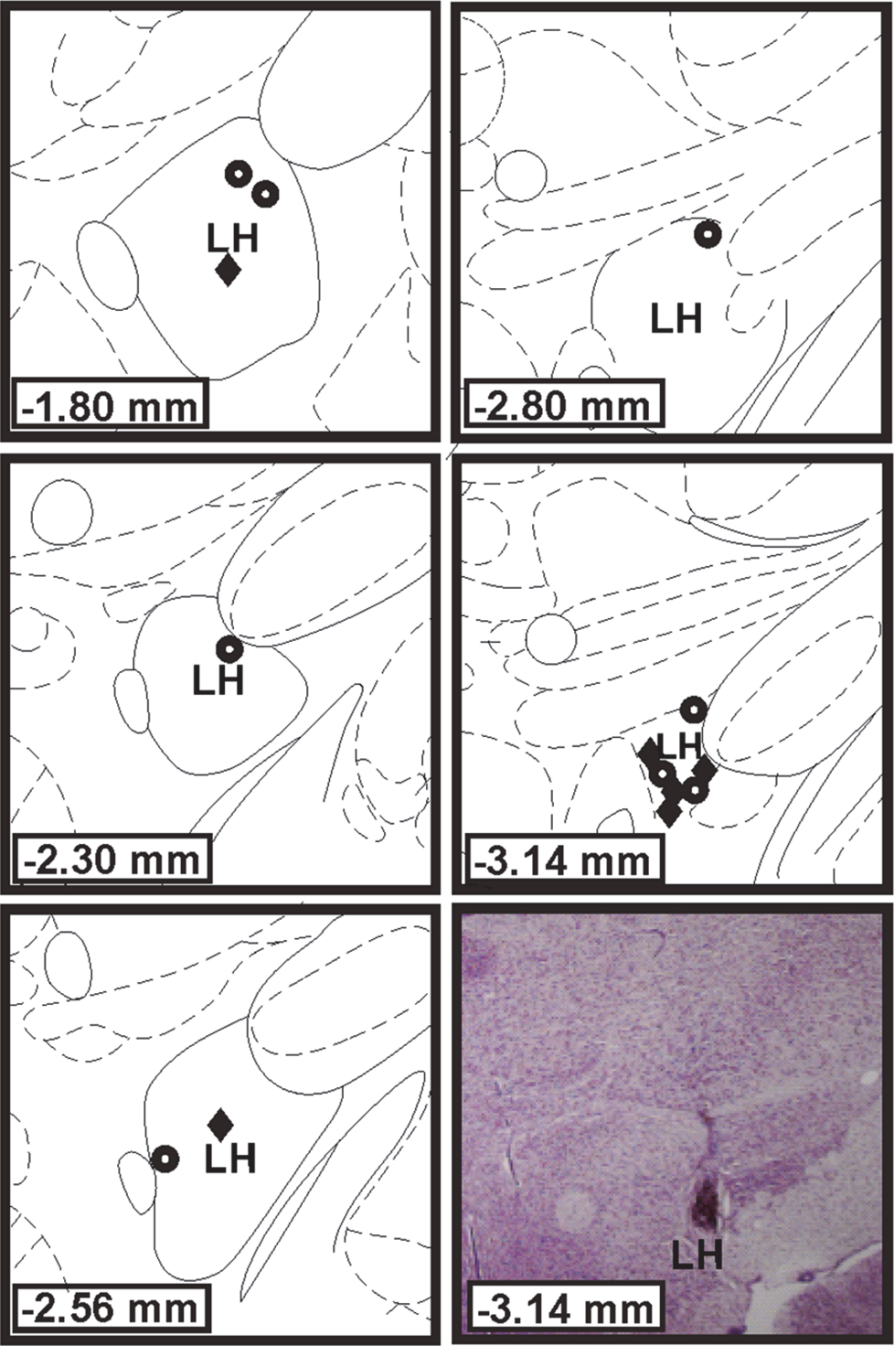
Electrode tip placement for intracranial self-stimulation. Illustration of electrode tip locations targeted at the medial forebrain bundle at the level of the LH. Neuroanatomical illustrations were modified from Paxinos and Watson (1998) and numbers indicate distance from bregma (mm). The bottom right panel shows a high magnification photomicrograph of a LH coronal section stained with cresyl violet. This section represents an electrode that was implanted for 90 days. Circles indicate sham controls whereas diamonds indicate lesioned rats. LH, Lateral hypothalamus.

Tip locations for the stimulating electrodes were determined *post mortem*. All rats that performed ICSS had electrode tips that were located within the lateral hypothalamus of the medial forebrain bundle (Fig 4). The average current amplitudes necessary to elicit optimal responding for both lesioned and sham rats were 203.3±0.5μA and 222.2±0.4μA, respectively. These were not significantly different (*t*_(13)_ = 0.55, *p* = 0.59) suggesting that lesion state did not affect BrS parameters necessary for lever-press responding.

#### Phase 1. Shaping

Lesioned and sham rats met the criteria for this phase (i.e., >8 lever presses/min) in an average of 2.5±0.3 and 2.3±0.2 test sessions, respectively. Task acquisition did not differ between treatment groups (*t*_(13)_ = 0.49, *p* = 0.63).

#### Phase 2. FR-1 Schedule of Reinforcement

**Fig 5:**
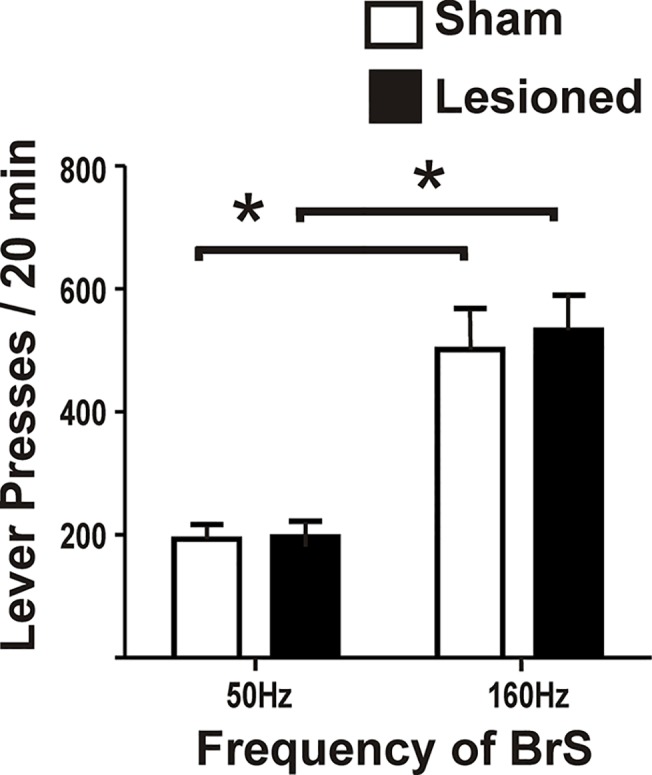
Rats lever press in a fixed ratio-1 reinforcement test at increased rates for the large reinforcer (LR; 160Hz) over the small reinforcer (SR; 50Hz). Rats lever pressed for current stimulation frequencies at 50Hz and 160Hz. Both frequencies met our criteria for stable responding (8< lever presses/ min). These data demonstrate that 50Hz is sufficient to elicit moderate lever press responding and furthermore, 160Hz is more reinforcing than 50Hz as shown in the number of lever presses per session. These frequencies were used for the small (50Hz) and large reinforcer (160Hz) in the discounting task (Student’s *t*-test, p<0.05).

Lesioned and sham rats met the criteria for this phase (i.e., self-initiated lever pressing and maintenance of stable responding) in an average of 3.2 and 3.1±0.3 test sessions, respectively. Task acquisition did not differ between treatment groups (*t*_(13)_ = 0.14, *p* = 0.89). Average lever pressing rates for the final two consecutive sessions were not statistically different between groups: left lever (lesioned = 351±40; sham = 356±44; t_(13)_ = 0.09, *p* = 0.93), right lever (lesioned = 336±33; sham = 368±36; t_(13)_ = 0.61, *p* = 0.55). Lever pressing rates were also not different between the two levers within either group, indicating an absence of a bias (lesioned, t_(10)_ = 0.28, *p* = 0.69; sham, t_(16)_ = 0.21, *p* = 0.83). To confirm that the frequencies selected for the SR and LR were reinforcing, a subset of lesioned and sham rats (lesioned,6; sham, 6) were tested in two consecutive FR-1 sessions for both 50Hz and 160Hz (Fig 5). There was no difference in lever pressing rates at either the large or the small current frequency (used for LR and SR, respectively) between lesioned and sham rats (50Hz; *t*_(10)_ = 0.13, *p* = 0.89; 160Hz; *t*_(10)_ = 0.36, *p* = 0.73). Both groups showed higher lever pressing rates at 160Hz (LR) compared to 50Hz (SR) (lesioned; *t*_(5)_ = 5.99, *p* = 0.001; sham; *t*_(5)_ = 4.51, *p* = 0.006). Lever pressing for the SR met our criteria for stable responding (>8 presses/minute) indicating that the SR was sufficient to maintain stable lever press responding. With these standardized BrS parameters, all rats reliably discriminated the 50Hz- from the 160Hz-designated levers, i.e., at the 0s delay, rats preferred the LR over the SR ~85% of the time. The preference for the LR in this choice test verified that the ICSS value used for the LR was more reinforcing than the ICSS value used for the SR.

#### Phase 3. Delay discounting task

**Fig 6:**
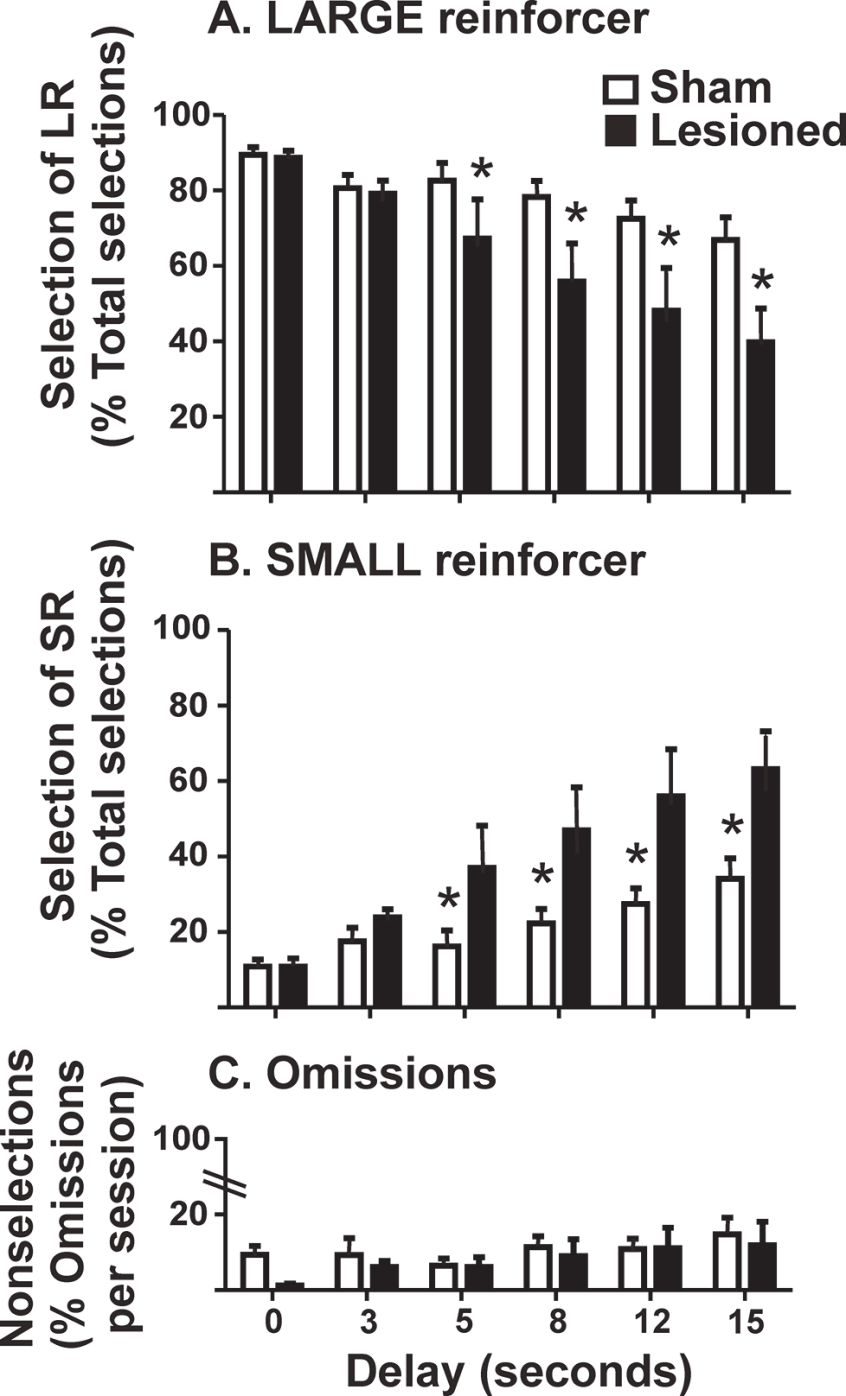
Lesioned rats show increased discounting behavior in the delay discounting task. Sham control (n = 9) and lesioned (n = 6) rats were tested once-daily in single session delays until stable behavior was observed (< 20% variability) upon which they were incremented to the next delay in ascending order. These rats include those which completed the entire task with the same history of delay presentation. Shown is the free-choice ratio (i.e., the number of selections made for LR/SR divided by total selections x 100). **(A)** Both groups exhibited preference for the LR when the imposed delay was small but lesioned rats progressively decreased preference for the LR when the delay was increased. **(B)** Choice of the SR increased as delay length was increased. **(C)** Rats exhibited low omissions throughout the delays tested in the discounting paradigm. Shown are the percent omissions (# omissions averaged for free-choice trials divided by total trial # x 100). Data are presented as mean + SEM (two way rmANOVA; *post hoc* Newman-Keuls, * p<0.05).

Two rats (1 sham and 1 lesioned) lost their head-stages during the behavioral task; therefore, they were omitted from further study. The remaining rats (lesioned, n = 13; sham, n = 14) initially preferred the delayed, LR; however, as the delay was increased, preference for the LR decreased and selection of the SR lever increased. At the longer delays, this effect appeared to be enhanced in lesioned rats (data not shown). To statistically verify this qualitative conclusion, we conducted a repeated measures analysis on rats that met criteria for stable behavior that completed all delays ranging from 0–15s with the same delay presentation history (i.e., lesioned, 9; sham, 6) (Fig 6). Significant effects were obtained for lesion status [*F*_1,13_ = 5.696, p = 0.03], delay [*F*_5,65_ = 16.32, p<0.001], and an interaction [*F*_5,65_ = 3.02, p = 0.02]. A *post hoc* Newman-Keuls revealed lesion state differences at 5, 8, 12 and 15s delays, wherein lesioned rats shifted preference from the delayed LR to the immediate SR at shorter delays than did the shams. These results indicate that when the delays were brief, both lesioned and sham rats preferred the delayed LR; however, as delays were increased, preference for the LR progressively decreased, and this effect was greater in lesioned rats.

The number of omissions throughout the discounting test session was monitored and it was determined that omissions constituted ~8.5% of free-choice trials across all delays of 0–15s. Based upon the percent omissions obtained from the free-choice trials during the final three consecutive sessions that met criteria for stable behavior, there was no effect of lesion status [*F*_1,13_ = 1.07, p>0.05], no effect of delay [*F*_5,65_ = 1.58, p>0.05] or an interaction [*F*_5,65_ = 0.37, p>0.05] indicating that the reported discounting results were not influenced by omissions during the discounting task. The number of omissions increased considerably with delays extending past 15s (>50%), which would have interfered with proper interpretation of the data; therefore, these data were not included in any of the analyses. To verify that discounting results were not confounded by habitual and/or compulsive responding on any one particular lever, we assessed the % selection of the LR when the LR was associated with the left versus the right lever. Rats showed no significant difference in LR selection between levers (refer to S2 Fig for data collected from the 5s delay) indicating that habitual responding is not likely to influence choice behavior.

To capture the capacity of the rats to transfer the learned association between lever pressing and BrS when a delay is imposed between the action and the positive reinforcement, a subset of sham rats (n = 4) that exhibited stable LR responding of ≥80% the 0s delay were subsequently tested with a 15s delay. These rats exhibited high omission rates at the 15s delay (>50%; 0 delay *vs*. 15s delay; *t*_(3)_ = 7.32, *p* = 0.005) (S3 Fig). Rats were retested with the 0s delay where they re-established stable performance (<10% omissions) and were subsequently tested with an incremental delay presentation of 3, 5, 8, 12 and 15s. This approach resulted in few omissions at 15s delays (>20%) and provided rationale for utilizing the ascending delay paradigm in the study.

## Discussion

The present report describes an ICSS-mediated delay discounting task for rats and reveals that dopaminergic inputs to the DLS regulate delayed reinforcement. Each of these findings is discussed below.

We previously demonstrated that ICSS supports *probability* discounting in rats [24–26]; here, we show that ICSS can be used in a *delay* discounting task. The rats quickly learned to lever press for reinforcing BrS and were able to differentiate between a small ICSS reinforcer, 50Hz, and a large ICSS reinforcer, 160Hz. Using these standardized SR and LR values, rats were tested in the discounting task with delays that ranged from 0–15s. Both lesioned and sham rats showed high preference for the LR when the delay was relatively short. As the LR delays were extended, rats progressively increased preference for the immediate SR. Habitual responding did not influence choice behavior as selection for the LR remained unchanged regardless of which lever was associated with the LR. These discounting results are consistent with those obtained across species, and with alternate reinforcement paradigms for discounting tasks [4,28].

In the current study, trained rats were gradually introduced to sequentially longer delays, and with this procedure we obtained stable performance with low omissions up to a 15s delay. A pilot study demonstrated high omission rates (>50%) in sham rats tested at delays extending past 15s and therefore longer delays were not assessed. At the longest delay tested using the incremental approach (i.e., 15s), sham rats showed a 27% shift from the delayed LR to the immediate SR. This shift in magnitude was sufficient to reveal that discounting behavior increased following dopaminergic lesions of the DLS. As we did not observe a steeper discounting effect in our sham control rats at the longer delays tested, this task may be limited in its utility to reveal robust *decreases* in discounting behavior (i.e., increased preference for the delayed LR). The order of presentation for the delays was relevant for this task. High omission rates occurred when rats were tested in these delays presented in *descending* order, i.e., when rats were tested in the 15s delay following the 0s delay. When rats were reintroduced to the 0s delay and subsequently tested with incremental delays of 3, 5, 8, 12 and 15s, omissions remained low even for the longer delays. This outcome indicates that the magnitude of change in delay length during task acquisition contributed to the tolerance level for a given delay, or that the rats were not able to transfer the learned association between pressing the LR lever and the delivery of the LR when large differences in delay were imposed between sessions. These optimal delays for ICSS reinforcement differ from those traditionally used in food-reinforced paradigms, wherein delays typically extend out to lengths of 1min. This procedural difference likely reflects the robust and instantaneous nature of ICSS; in contrast, food reinforcement requires several intermediary steps, including obtainment, mastication and ingestion. Reinforcer delivery (e.g., BrS) strengthens its association with a given action (e.g., lever press) and these associations can be disrupted when the reinforcer is significantly delayed after the action is executed [29]. Some studies suggest that ICSS efficacy degrades more rapidly than conventional positive reinforcers as the time between behavioral response and reinforcer delivery is increased [30–32]. Repetitive training in FR-1 schedules of reinforcement in phases 1 and 2 of the test paradigm strengthens the relationship between lever-press responding and BrS reinforcement. This enhanced association allowed for rats to acquire and successfully execute the discounting task even in the presence of reinforcer delays up to 15s.

We used the ICSS-mediated paradigm to determine the effects of dopaminergic lesions of the DLS on delay discounting behavior. 6-OHDA-induced lesions of the DLS resulted in near complete loss of dopaminergic terminals in the DLS and a significant decrease in dopaminergic cell bodies in the SNpc. Motor stepping deficits were used to functionally verify the lesion as these deficits are comparable to the postural instability and akinetic behavior displayed by humans with similar brain-states (e.g., individuals with early-stage Parkinson’s disease, which reflects a reduction in nigral dopaminergic neurons and a large loss of dopaminergic terminals within the putamen) [33], the human homolog of the rat DLS. It is noteworthy that the lesion state did not impair the ability of rats to perform in the operant task as lever-press responding in the FR-1 test and acquisition of each phase of the discounting task did not differ between lesioned and sham groups. Discounting was significantly enhanced in the lesioned group at the 5, 8, 12, and 15s delays compared to shams. These findings revealed that dopaminergic innervation of the DLS is necessary for normal discounting, and support a role for dopaminergic inputs to the DLS in impulsive choice behavior. As reward valuation did not differ between lesioned and sham rats at either 50Hz and 160Hz (i.e., the treatment groups were not different in lever-press responding for the SR or LR in an FR-1 schedule of reinforcement), it is unlikely that deficits in reward processing in lesioned rats impacted choice behavior. These findings add to the growing literature that supports a role for dopaminergic transmission in delay-based decision-making. For example, dopamine antagonists increase discounting behavior in several variations of these tasks in rats [34–37]. In addition, increases in reaction time errors are seen at short and medium delays during an impulsivity task in monkeys where dopaminergic systems were lesioned with MPTP [38]. Regarding the role of the DLS, it appears that the nature of the DLS lesion is a critical feature of the resultant effect on impulsive choice, for delay insensitivity (i.e., *decreased* discounting) is reported for rats with kainic acid-induced lesions of neurons within the dorsal striatum (lateral plus medial aspects) [39]. Intra-striatal injections of excitotoxins, like kainic acid, lesion neuronal somata, leaving inputs and fibers of passage largely intact. In contrast, 6-OHDA infusions (preceded by ip desipramine) result in selective dopamine deafferentation, and this would mostly impact the subset of DLS neurons that are post synaptic to dopaminergic inputs. Thus, the DLS clearly plays a critical role in impulsive decision-making during delayed reinforcement, and the nature of this role depends on a balance between dopaminergic influences and DLS neurons that are not post synaptic to these inputs.

We previously determined that dopamine deafferentation of the rat DLS does not alter ICSS-mediated *probability* discounting, wherein rats choose between a certain SR and a LR delivered after uncertain probabilities [26]. While probability and delay discounting share overlapping learning and decision-making processes, the current study indicates that features which are unique to delay discounting are more sensitive to the status of DLS dopamine. One such feature is the temporal component in delay discounting tasks. Time perception is tightly coupled with delay discounting as the subjective value of larger, delayed rewards is reduced as a function of increased delay time. Indeed, it has been suggested that deficits in time perception may contribute to the steeper discounting seen in more impulsive individuals [4,40]. The range of delays often tested in delay discounting tasks falls within a temporal processing timeframe referred to as interval timing. Interval timing relates to the capacity to accurately perceive the passage of time within the seconds to minutes range, and individuals suffering from striatal neuropathologies (e.g., Parkinson’s disease) are known to show deficits in time perception [41]. Time estimation studies in humans with Parkinson’s disease show overestimation in elapsed time intervals in the millisecond to several minutes range, indicative of a slowed internal clock [42–47]. Further evidence indicating a role for the putamen in temporal processing is provided by Nenadic and colleagues [48], who used fMRI to study brain connectivity during a time estimation task in healthy human volunteers. Using a task that controlled for time discrimination, attention, and decision-making, they found that the putamen is strongly associated with perceptual timing. Research on non-human laboratory animals has established that dopamine agonists and antagonists decrease and increase time estimations, respectively [49–52]. Dopaminergic lesions of the dorsal striatum in rats induce robust impairment of performance in timed estimation tasks demonstrating a role for the dorsal striatum in temporal processing [53]. Heilbronner and Meck [54] found that cocaine and methamphetamine increase impulsive choice as well as perceived clock speed (i.e., decreases in time estimation). These findings suggest that changes in both time perception and delay discounting occur with increased dopamine transmission. Impulsive choice is contingent on the ability to accurately estimate the perception of elapsed time prior to reward delivery. Thus, if brain circuitry regulating time perception are dysregulated, it is likely that impulsive choice behavior becomes dysfunctional as well. Further studies are needed to determine the particular behaviors that drive delayed discounting.

The observed outcomes may also reflect the emotional status of the subjects. For example, apathy, described as a reduction in self-generated, purposeful behavior, is suggested to result from hypodopaminergic brain-states [55]. A significant subset of untreated early-stage Parkinson’s disease patients (<70%) report apathy indicating that loss of dopamine innervation of the striatum may contribute to this behavior [56]. One component of apathy includes the impaired ability to foresee future rewards [57]. Thus, apathy may reduce the motivational drives toward optimal decision-making in delay discounting tasks by disrupting option selection associated with delayed reward outcomes thereby driving preference for immediately available rewards. Recent studies demonstrate that dopamine deafferentation of the dorsal striatum in rats produces profound motivational deficits during reward-related operant responding [58]. Loss of motivational functions of the dorsal striatum has been proposed as a putative mechanism underlying apathy in PD patients [55].

## Conclusions

In summary, we have developed a novel ICSS-mediated protocol for measuring delay discounting in rats; a putative index of impulsive choice. We found that dopaminergic lesions of the DLS decreased preference for the delayed LR at a faster rate than sham controls; thus, discounting was enhanced. These data add to a growing body of evidence for the contribution of the DLS to govern impulsive choice, one aspect of impulsive decision-making. Evidence suggests that striatal dysfunction impairs time estimation, potentially rendering the individual delay-intolerant, and may also contribute to apathy. These behavioral deficits provide several avenues through which discounting behavior may be influenced by dopaminergic lesions of the DLS. Studies focusing on discriminating neural circuitry and transmitter systems involved in time perception, apathy and delay discounting tasks as well as the potential overlap between these processes would aid in determining whether alterations in delay-based decision-making are measuring impulsivity *per se*, or additionally reflect deficits in temporal processing and/or apathetic drives.

## Supporting Information

S1 Fig Current stimulation frequency vs. lever-press response curve. A separate cohort of sham rats (n = 9) were used to determine standardized values for the small reinforcer (SR) and large reinforcer (LR) in the discounting task. Rats were trained in a FR-1 schedule of reinforcement to press for current frequencies ranging from 10–160Hz. Each frequency was presented for 2min and the order of presentation was randomized. Data are shown from the last three consecutive sessions with <20% variability. Rats demonstrated increased lever-press response rates as current frequency was increased. 50Hz was selected for the SR, which was above threshold for lever-press responding and 160Hz was selected for the LR, which induced near maximal responding. Data are presented as mean ± SEM.
(TIF)

S2 Fig Selection of LR is independent of the associated lever. The data collected during the discounting tasks for sham (n = 9) and lesioned (n = 6) rats were analyzed to determine if lever bias, or habitual responding, influenced % selection of the LR. Data shown are the last two sessions of stable behavior during the 5s delay where the LR was associated with either the left or right lever. Sham and lesioned rats show no change in selection of the LR. Data are presented as mean + SEM.
(TIF)

S3 Fig Effect of delay history on omission rate in the delay discounting task. Following stable behavior at the 0s delay (<20% variation over 3 consecutive sessions), a subset of sham rats (n = 4) were tested in the 15s delay. This behavioral protocol increased trial non-selections (i.e., omissions observed in the free-choice trials). Rats were returned to the 0s delay where performance was re-established. Using the incremental approach (i.e., 3, 5, 8, 12, and15s) rats demonstrated low numbers of non-selections following re-introduction of the 15s delay. Data are presented as mean + SEM. *Student’s t-test*, * *p<0*.*05*.
(TIF)

S1 Text Included in the text are additional details regarding the implementation of intracranial-self stimulation in a delay discounting task. (DOCX)
