# Supplementary figures and images for: Dopaminergic Lesions of the Dorsolateral Striatum in Rats Increase Delay Discounting in an Impulsive Choice Task
Source: PLoS One. 2015 Apr 30;10(4):e0122063. doi: 10.1371/journal.pone.0122063 (PMC4415807; doi:10.1371/journal.pone.0122063)

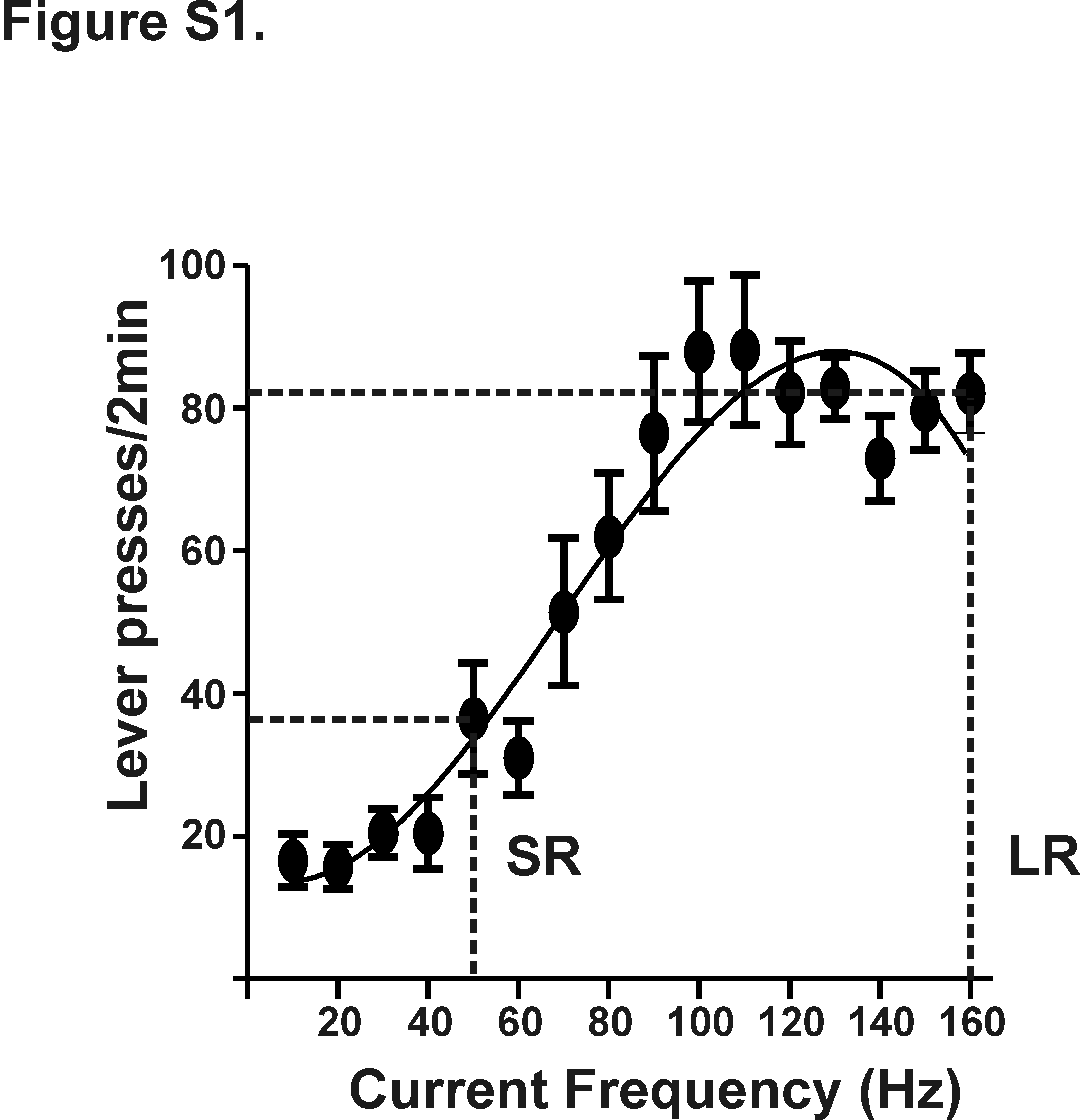

Supplement: S1 Fig — A separate cohort of sham rats (n = 9) were used to determine standardized values for the small reinforcer (SR) and large reinforcer (LR) in the discounting task. Rats were trained in a FR-1 schedule of reinforcement to press for current frequencies ranging from 10–160Hz. Each frequency was presented for 2min and the order of presentation was randomized. Data are shown from the last three consecutive sessions with <20% variability. Rats demonstrated increased lever-press response rates as current frequency was increased. 50Hz was selected for the SR, which was above threshold for lever-press responding and 160Hz was selected for the LR, which induced near maximal responding. Data are presented as mean ± SEM. (TIF) [file pone.0122063.s001.tif]

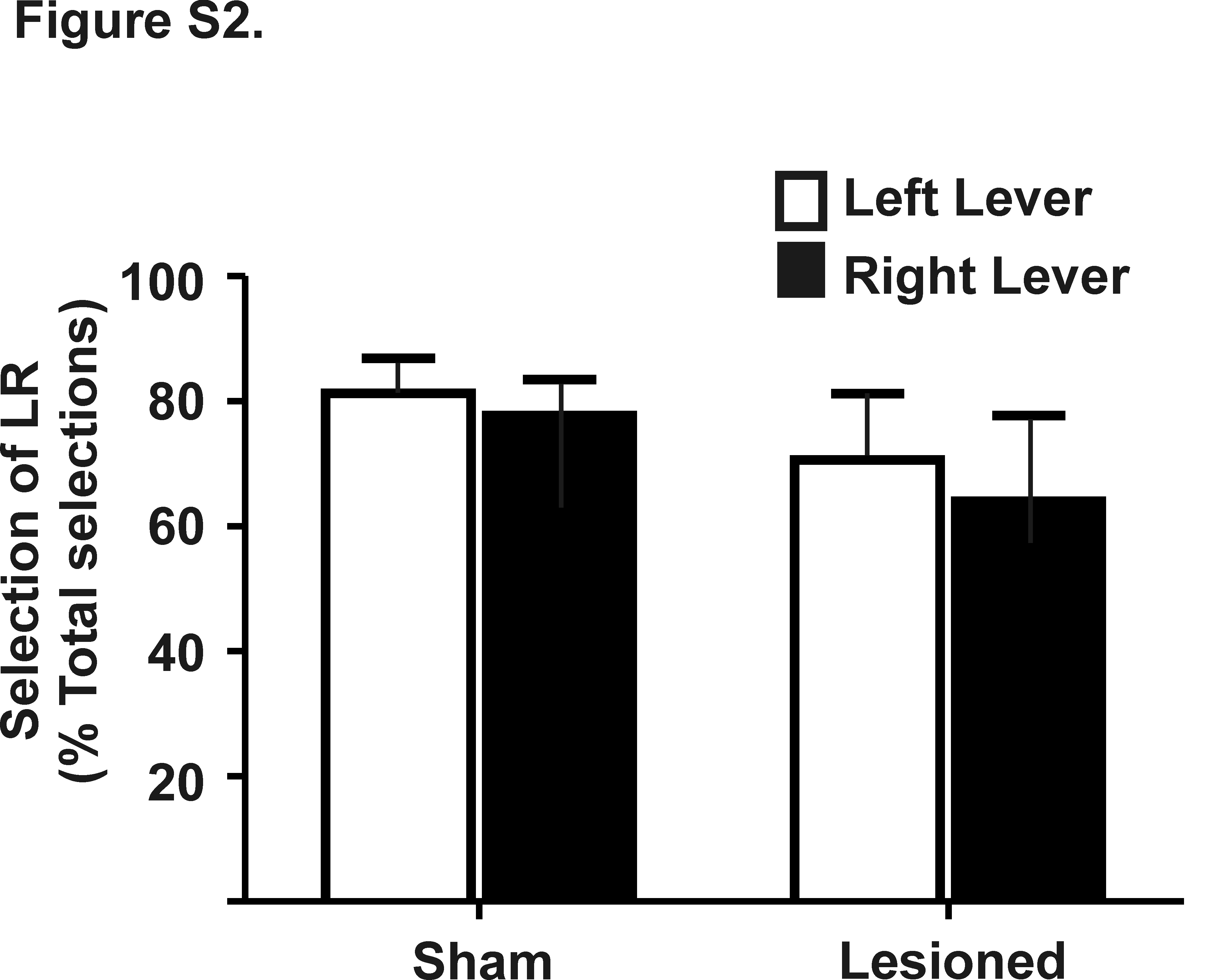

Supplement: S2 Fig — The data collected during the discounting tasks for sham (n = 9) and lesioned (n = 6) rats were analyzed to determine if lever bias, or habitual responding, influenced % selection of the LR. Data shown are the last two sessions of stable behavior during the 5s delay where the LR was associated with either the left or right lever. Sham and lesioned rats show no change in selection of the LR. Data are presented as mean + SEM. (TIF) [file pone.0122063.s002.tif]

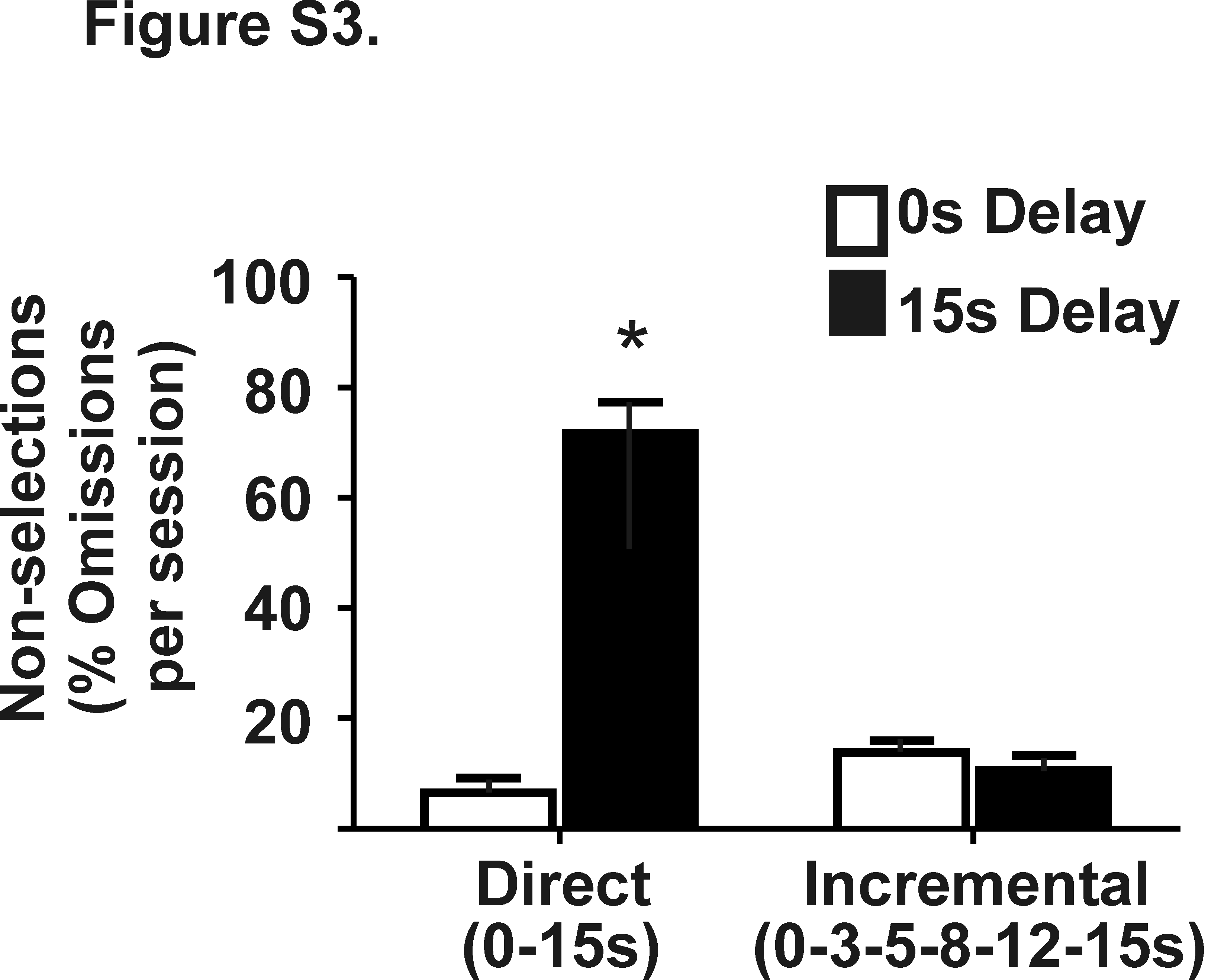

Supplement: S3 Fig — Following stable behavior at the 0s delay (<20% variation over 3 consecutive sessions), a subset of sham rats (n = 4) were tested in the 15s delay. This behavioral protocol increased trial non-selections (i.e., omissions observed in the free-choice trials). Rats were returned to the 0s delay where performance was re-established. Using the incremental approach (i.e., 3, 5, 8, 12, and15s) rats demonstrated low numbers of non-selections following re-introduction of the 15s delay. Data are presented as mean + SEM. Student’s t-test, * p<0.05. (TIF) [file pone.0122063.s003.tif]
