# Supplementary material for: Dopaminergic Lesions of the Dorsolateral Striatum in Rats Increase Delay Discounting in an Impulsive Choice Task
Source: PLoS One. 2015 Apr 30;10(4):e0122063. doi: 10.1371/journal.pone.0122063 (PMC4415807; doi:10.1371/journal.pone.0122063)
Supplement: S1 Text — (DOCX) [file pone.0122063.s004.docx]

**Supporting Information**

**Procedures for intracerebral injections of the dopaminotoxin and implanting stimulation electrodes**

Rats were habituated to the vivarium for a minimum of seven days and handled at least three times prior to surgical procedures. Rats were given an intraperitoneal injection of desipramine-HCl (25mg/kg as the salt) (Sigma-Aldrich, St Louis, MO) dissolved in sterile water 30 min prior to the lesion to reduce uptake of the 6-OHDA into adrenergic neurons. Rats were anesthetized using isoflurane (delivered at a concentration of 1-3% in oxygen) and placed in a stereotaxic apparatus (nosepiece set at -3.3mm below the horizontal; David Kopf, Tujunga, California). 6-OHDA (Sigma-Aldrich) was dissolved in 0.2% ascorbic acid in a sterile saline solution (pH=5.0) and injected bilaterally into the striatum at a dose of 7.5μg (as the salt) per 2μl per side at a rate of 0.2µl/min for 10min. Sham-lesioned rats were injected with the ascorbic acid vehicle. The cannula was left in place for an additional 1 min to allow diffusion from the injector tip, and the burr holes were filled with bone wax.

A bipolar stimulating electrode (MS303/3-B/SPC; Plastics One, Roanoke, VA) was implanted in the lateral hypothalamus. Rats were implanted in both the left and right hemisphere with no observed change in ICSS responding or behavioral bias. Therefore, we opted to implant exclusively in one hemisphere, i.e., right hemisphere. The electrode was secured to the skull with five stainless steel screws and dental acrylic. The incision was sutured, and rats were allowed to recover for a minimum of five days before behavioral testing began.

**Verification of electrode placement and 6-OHDA lesion**

Upon completion of the behavioral assessments, the rats were euthanized with carbon dioxide, brains were extracted, immersion-fixed in 10% buffered formalin for 24h, and stored in 30% sucrose at 4^o^C until completely saturated. Brains were sliced into serial coronal sections (40µm) using a sliding microtome and stored in cryoprotectant at -20°C. Sections containing the lateral hypothalamus were mounted on slides and stained with cresyl violet (a Nissl stain) to aid in visualization of the electrode tip location. The location was verified by at least two observers and mapped onto standardized coronal sections of the brain.

The lesion produced by intra-striatal injections of 6-OHDA was determined using immunohistochemical (IHC) staining for tyrosine hydroxylase (TH) [2]. In brief, brain slices were rinsed in a Tris-buffered saline (TBS) solution containing triton-X, and endogenous peroxidase activity was quenched by incubation in 0.1M sodium periodate in TBS for 20min. Non-specific staining was blocked by incubation in phosphate-buffered saline containing 3% normal serum and 2% bovine serum albumin. Sections were then incubated in primary antibody (1:10,000; ImmunoStar, Hudson, WI) overnight at room temperature. Following thorough rinsing, sections were incubated with biotinylated secondary antibody (horse anti-mouse, rat adsorbed, 1:200; Vector Laboratories, Burlingame, CA) for 1h. The signal was amplified by avidin /biotin-based peroxidase using the Elite ABC Vectastain Kit (1:500; Vector Laboratories). Immunostaining was visualized using a chromogenic solution containing 0.5% 3,3-diaminobenzidinetetrachloridedihydrate (Sigma-Aldrich) and 1% H_2_O_2_. Lesion extent within the striatum was characterized by a near absence of TH-like staining as agreed upon by at least two observers, and diagramed to both hemispheres in standardized coronals sections of the forebrain. To determine if pathology in striatal dopaminergic terminals extended to the cell bodies in the lateral aspects of the Substantia nigra pars compacta (SNpc), we quantified the number of TH^+^ soma using stereological approaches. To do so, an Olympus BX60 microscope with a computer-controlled motorized stage, high sensitivity HV-C20 CCD video camera (Hitachi, Japan) and StereoInvestigator software version 5.1(MicroBrightField, Colchester, VT) was used to analyze randomized equidistant serial sections of individual rats. The number of TH^+^ cells was counted within the thickness of the tissue section under an Olympus 100× objective using unbiased three-dimensional counting. A total of five sections containing the SNpc were quantified per animal, and approximately 20% of the region was assessed using a 70x70µm counting frame. The total number of TH^+^ cells and the volume of tissue evaluated were divided to obtain a density measurement expressed as total number/mm^3^.

**Behavioral Task: Motor**

To validate the 6-OHDA-induced DLS lesion, we used the forelimb adjustment step test, as employed previously [3]. Tests were conducted prior to surgery, and at several time points after surgery, throughout the behavioral paradigm. The rat was moved laterally over a distance of 0.9m for 5s in the abduction direction and the number of steps the rat displayed to adjust his weight was counted. Three trials were taken per session, and the average score was determined. Left and right forelimb stepping was similar and results are presented using only data collected from the right forepaw.

**Behavioral Task: Delay Discounting**

ICSS studies were performed based upon our previously established protocols [2-3]. Rats were trained in standard rat operant chambers (30.5cm×24.1cm×21.0cm) equipped with two retractable levers located 5cm above the grid floor (Med-Associates, St. Albans, VT). A 100mA stimulus light was located above each lever and a single 100mA house light was located in the top center of the chamber wall opposite the levers. During each test session, the house light was illuminated while the lever(s) were extended indicating the opportunity for delivery of brain stimulation (BrS) to the stimulating electrode. A three-phase delay discounting paradigm was designed to measure changes in impulsive choice.

**Phase 1, shaping.** A single lever was extended inside the operant chamber. BrS was delivered non-contingently upon forward movement toward the extended lever. Current intensity was started at 100µA and was adjusted for each rat in 20µA increments based upon each rat’s behavior to approach and subsequently press the lever. The final current intensity that produced high rates of lever pressing was used for the remainder of the paradigm.

**Phase** **2, fixed ratio-1 (FR-1) reinforcement.** Rats were trained in a FR-1 schedule of reinforcement to establish stable (>8 lever presses/min) lever pressing responses. One lever was extended throughout each 20 min session which upon a single lever press, one BrS was delivered.

**Phase 3, delay discounting task.** For this task, upon lever pressing, one lever delivered the small, immediate reinforcer (SR; 50Hz) and the other delivered a large reinforcer (LR; 160Hz) that was given after a delay ranging between 0-15s.

The standardized reinforcers used in the delay discounting task were determined based upon data collected from a current stimulation frequency vs. lever-press response curve (Figure S1). A separate group of sham rats (n=9) were tested in a FR-1 schedule of reinforcement at frequencies ranging from 10-160Hz. Frequencies were available for 2min bins and were presented in randomized order. According to these population frequency curves, 50Hz elicited stable, although moderate, lever pressing and160Hz was close to frequencies that elicited maximal lever pressing. These values were therefore used as the SR and LR, respectively. The delays preceding delivery of the LR were 0, 3, 5, 8, 12 and 15s. Test sessions were conducted with only one delay at a time. Following stable behavior (<20% variability over three consecutive sessions), rats were advanced to the subsequent delay, in ascending order. To standardize free-choice trial length between selections that resulted in preference for the immediate SR verse the delayed LR, trial length was dictated based on the total time necessary to obtain the delayed LR, regardless of selection (i.e., each trial comprised of a 10s selection opportunity between the immediate SR and the delayed LR and the subsequent delay time necessary for the delayed reinforcer delivery). For example, a full trial in a 3s delay task totals 13s. (10s for the opportunity to select, and 3s to include the time necessary to deliver the 3s delayed LR). For a 15s delay trial the total trial length is 25s (10s for the opportunity to select and 15s to include the time necessary for the delayed reinforcer delivery). Following each full trial is a 10s inter-trial timeout. The decision to test only one delay per day was based on a pilot study wherein rats were tested in a within session change of delay ranging from 3-15s (i.e., 3, 5, 8, 12 and 15s) in ascending order. This protocol resulted in a high omission rate (>50% of free-choice trials) in the majority of rats tested, such that responding fell below our minimum criterion for inclusion of data into analysis. Longer delays have been assessed using food-reinforced delay discounting paradigms without significant omission rates. Differences in optimal delay ranges used in food-reinforced *vs.* ICSS-reinforced paradigms may relate to the robust and instantaneous nature of ICSS [4].

**References**

1. Paxinos G, Watson C (1998): *The Rat Brain in Stereotaxic Coordinates.* Academic Press: New York.

2. Rokosik SL, Napier TC (2011) Intracranial self-stimulation as a positive reinforcer to study impulsivity in a probability discounting paradigm. J Neurosci Methods 198: 260-269. doi: 10.1016/j.jneumeth.2011.04.025..

3. Rokosik SL, Napier TC (2012) Pramipexole-induced increased probabilistic discounting: comparison between a rodent model of Parkinson's disease and controls. Neuropsychopharmacology 37: 1397-1408. doi: 10.1038/npp.2011.325.

4. Tedford SE, Holtz NA, Persons AL, Napier TC (2014) A new approach to assess gambling-like behavior in laboratory rats: using intracranial self-stimulation as a positive reinforcer. Front Behav Neurosci 8: 215. doi: 10.3389/fnbeh.2014.00215
